# Native valve endocarditis due to *Escherichia coli* infection: a case report and review of the literature

**DOI:** 10.1186/s12872-018-0929-7

**Published:** 2018-10-19

**Authors:** Nobuhiro Akuzawa, Masahiko Kurabayashi

**Affiliations:** 1 Department of General Medicine, National Hospital Organization Shibukawa Medical Center, 383 Shiroi, Shibukawa, Gunma 377-0280 Japan; 2 Department of Medicine and Biological Science, Gunma University Graduate School of Medicine, 3-39-22 Showa-machi, Maebashi, Gunma 371-8511 Japan

**Keywords:** *Escherichia coli*, Excessive alcohol consumption, Infective endocarditis, Purulent spondylitis

## Abstract

**Background:**

Infective endocarditis due to *Escherichia coli* is a rare disease but is increasing in frequency, especially among older women. In addition, its mortality rate is higher than that of endocarditis due to the HACEK-group gram-negative bacteria (*Haemophilus* spp., *Aggregatibacter* spp., *Cardiobacterium hominis*, *Eikenella corrodens*, and *Kingela* spp.).

**Case presentation:**

A 58-year-old Japanese woman with a history of alcohol abuse was admitted to our hospital because of a fever. She was diagnosed with infective endocarditis due to *E. coli* based on repeated blood cultures and transthoracic echocardiography, which revealed vegetations attached to the anterior leaflet and chordae tendineae of the mitral valve. Despite administration of sulbactam/ampicillin and gentamycin, she developed purulent spondylitis during hospitalization and required treatment with meropenem administration for 6 weeks, leading to resolution of the endocarditis. She took oral levofloxacin for 2 months, and the spondylitis was completely cured 7 months after discharge.

**Conclusion:**

*Escherichia coli* affects native valves without degenerative valvulopathy rather than prosthetic valves, especially in patients with risk factors such as an immunosuppressive status, excessive alcohol consumption, or treatment with hemodialysis. Peripheral embolization, congestive heart failure, and valve-ring abscesses are major complications of *E. coli* endocarditis; notably, infective myocarditis can also occur. The mortality and surgical intervention rates are 21% and 42%, respectively. Physicians should be cognizant of the necessity of surgical intervention when *E. coli* endocarditis is resistant to antibiotic therapy.

## Background

Infective endocarditis (IE) due to *Escherichia coli* is a rare disease. *Escherichia coli* is the causative microorganism in approximately 0.51% of cases of IE [1]. Thirty-six cases of *E. coli* native valve IE that met the Duke criteria were reported in the literature from 1909 to 2002, and urinary tract infection was the most common cause of endocarditis due to *E. coli* [2]. The low incidence of *E. coli* IE has been attributed to the inability of this bacterium to adhere to the endocardium as well as the existence of antibodies to *E. coli* in normal serum [3]. Notably, however, the number of > 70-year-old patients with *E. coli* IE has recently increased, and about 70% of affected patients are older women [2]. In addition, the mortality rate of *E. coli* IE (21%) is higher than that of IE due to HACEK-group gram-negative bacteria (4%), including *Haemophilus* spp., *Aggregatibacter* spp., *Cardiobacterium hominis*, *Eikenella corrodens*, and *Kingela* spp. [1, 4].

We herein present a patient with native valve IE resulting from *E. coli* infection. This patient was admitted to our hospital for examination because of a fever of unknown origin. Repeated blood cultures revealed *E. coli*, but no *E. coli* grew in sputum or urine cultures. Transthoracic echocardiography demonstrated two vegetations adhered to the anterior leaflet of the mitral valve (MV) and its chordae tendineae. During hospitalization, the patient’s condition was complicated by purulent spondylitis. After 8 weeks of antibiotic administration, the patient was discharged without any sequelae.

## Case presentation

A 58-year-old Japanese woman was admitted to our hospital by ambulance because of a 1-week history of malaise, lumbago, and fever of unknown origin. She had no relevant medical history and no family history. She was a nonsmoker, but she had drunk about 60 to 80 g of alcohol per day for 30 years. Liver dysfunction had been noted for the past 10 years.

On admission, her height was 158 cm, weight 56.2 kg, and body temperature 39.5 °C. Her blood pressure was 101/60 mmHg. Her heart rate was 106 beats per minute. A physical examination showed no major abnormalities. She was alert but short of breath on exertion, with an arterial blood oxygen saturation of 94%, partial oxygen pressure of 72.0 mmHg, and partial carbon dioxide pressure of 27.2 mmHg. A chest radiograph showed no obvious signs suggesting pneumonia or pulmonary congestion. Electrocardiography showed sinus tachycardia and no other abnormalities. Plain computed tomography of the neck, chest, and abdomen and ultrasound of the abdomen revealed no significant abnormalities except for fatty change of the liver. Laboratory tests showed an elevated white blood cell count; elevated levels of serum liver enzymes, blood urea nitrogen, creatinine, uric acid, C-reactive protein (CRP), and serum brain natriuretic peptide; hypoproteinemia; and hypokalemia (Table 1). Serum rheumatoid factor was negative. Urinalysis was positive for ketone bodies, but the urinary sediment showed no abnormalities. Cultures of blood, urine, and sputum were carried out on admission. Intravenous administration of sulbactam/ampicillin (SBT/ABPC) (9 g/day) was begun from the day of hospitalization.

**Table 1:** Laboratory tests on admission

*Hematology*		Normal range
White blood cells^a^	11,400/mm^3^	3300–8600/mm^3^
Red blood cells	422 × 10^4^/mm^3^	386–492 × 10^4^/mm^3^
Hemoglobin	13.2 g/dL	11.6–14.8 g/dL
Hematocrit	41.2%	35.1–44.4%
Platelets	19.8 × 10^4^/mm^3^	15.8–34.8 × 10^4^/mm^3^
*Biochemistry*		
Total protein^a^	5.5 g/dL	6.6–8.1 g/dL
Albumin^a^	2.8 g/dL	4.1–5.1 g/dL
Total bilirubin	0.9 mg/dL	0.4–1.5 mg/dL
AST^a^	242 U/L	13–30 U/L
ALT^a^	127 U/L	7–23 U/L
LDH^a^	274 U/L	124–222 U/L
γ-GTP^a^	679 U/L	9–32 U/L
CPK	43 U/L	41–153 U/L
Amylase	45 U/L	44–132 U/L
Blood urea nitrogen^a^	38 mg/dL	8–20 mg/dL
Creatinine^a^	1.24 mg/dL	0.46–0.79 mg/dL
Uric acid^a^	9.0 mg/dL	2.6–5.5 mg/dL
Sodium	141 mEq/L	138–145 mEq/L
Potassium^a^	2.5 mEq/L	3.6–4.8 mEq/L
Chloride	102 mE/L	101–108 mEq/L
Glucose	102 mg/dL	73–109 mg/dL
Hemoglobin A1c	5.5%	< 5.8%
C-reactive protein^a^	22.5 mg/dL	< 0.15 mg/dL
BNP^a^	112.6 pg/mL	< 18.5 pg/mL
*Blood coagulation tests*		
PT-INR	1.02	0.85–1.15
*Serology*		
HBsAg	Negative	Negative
HCV-RNA	Negative	Negative
Anti-HAV IgM Ab	Negative	Negative
Anti-HIV Ab	Negative	Negative

*Abbreviations: AST* aspartate aminotransferase, *ALT* alanine aminotransferase, *LDH* lactate dehydrogenase, *γ-GTP* gamma-glutamyltranspeptidase, *CPK* creatine phosphokinase, *BNP* brain natriuretic peptide, *PT-INR* international normalized ratio of prothrombin time, *HBsAg* surface antigen of hepatitis B virus, *HCV-RNA* ribonucleic acid of hepatitis C virus, *Anti-HAV IgM Ab* anti-hepatitis A virus immunoglobulin M antibody, *Anti-HIV Ab* anti-human immunodeficiency virus antibody

^a^These values are outside the normal ranges

**Fig. 1:**
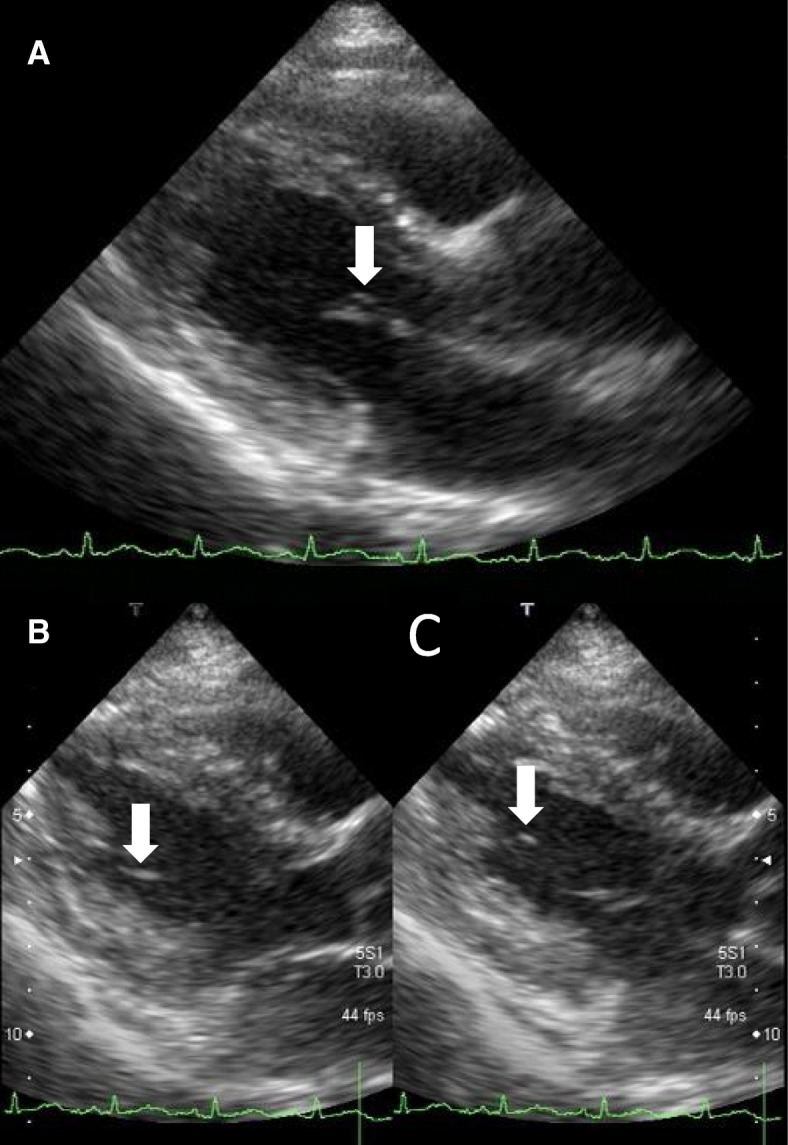
Transthoracic echocardiography images on day 2. **a**: A parasternal long-axis tomogram demonstrated a vegetation attached to the anterior leaflet of the mitral valve (4 × 1 mm, white arrow). **b, c:** Parasternal long-axis tomograms at (**b**) presystole and (**c**) end-diastole (white arrows.) A mobile and elongated vegetation (10 × 2 mm) was attached to the chorda tendinea of the mitral valve

On day 2, the patient remained febrile. The sputum culture showed no significant growth of pathogenic bacteria, and the urine culture was sterile. However, gram-negative rods were detected in the blood culture. Subsequent echocardiography revealed two vegetations, one of which was attached to the anterior leaflet of the MV and was approximately 4 × 1 mm in size (Fig. 1a). The other was attached to the chorda tendinea adjacent to the posterior papillary muscle, and its size was larger (10 × 2 mm) than that of the MV vegetation (Fig. 1b, c). Based on these findings, combined antibiotics including SBT/ABPC (9 g/day) and gentamycin (GM) (80 mg/day) were administered on day 2. Repeated blood cultures were also performed on days 2 and 3, and all culture sets were positive for gram-negative rods, which were proven to be pansensitive *E. coli* on day 6. Based on these findings, the patient was diagnosed with IE due to *E. coli* in accordance with the modified Duke criteria [5]. An ophthalmologist and dermatologist also examined her on day 6, but they observed no remarkable findings associated with IE. Contrast-enhanced computed tomography of the chest and abdomen on day 7 showed no remarkable findings; magnetic resonance imaging (MRI) and magnetic resonance angiography of the brain on day 8 also showed no abnormalities.

After antibiotic medication including SBT/ABPC and GM, the patient’s general condition improved. Her body temperature normalized on day 5, and her white blood cell count returned to a normal level (8600/mm^3^); her CRP level significantly decreased (5.09 mg/dL) on day 6. Blood culture analysis on day 7 and thereafter revealed no bacterial growth, but her CRP level remained mildly elevated (about 2.0 mg/dL). Echocardiography on day 14 showed a decrease in the size of the vegetations; those attached to the MV and chorda tendinea were 2 × 1 and 5 × 1 mm, respectively. However, the patient developed severe lumbago on day 20. Her white blood cell count was normal (5900/mm^3^), but her CRP level had increased to 3.1 mg/dL. MRI of the lumbar spine on day 21 revealed purulent spondylitis lesions in the L1 and L2 lumbar vertebrae. Intravenous meropenem (6 g/day) was substituted for SBT/ABPC and GM on day 21 despite the sterile blood culture. The patient’s clinical course was good thereafter; her lumbago improved. Transthoracic echocardiography on day 35 revealed complete resolution of the vegetations. On day 54, her CRP level decreased to 0.34 mg/dL. Administration of meropenem was terminated, and she was discharged from our hospital on day 56. After discharge, oral levofloxacin (500 mg/day) was administered for 2 months in accordance with the suggestions of orthopedic surgeons. Her CRP level normalized after 2 months. Both upper endoscopy and colonoscopy performed 3 months after discharge showed no abnormalities. MRI findings of the lumbar spine normalized after 7 months. She also stopped drinking alcohol for 12 months and remained well during that time.

## Discussion

IE due to *E. coli* is rare and most commonly observed in older women, especially those with diabetes mellitus [1]. However, IE due to *E. coli* can also be seen in younger patients. Fayyaz et al. [6] reported that *E. coli* was detected from 8.4% of 20- to 40-year-old patients with IE. They also reported that no more than 60% of detected *E. coli* showed susceptibility to amikacin and amoxicillin/clavulanate. Other studies have shown that *E. coli* accounts for one-third of non-HACEK, gram-negative bacilli-induced IE and is the most common gram-negative bacillus that can cause IE [7, 8]. These findings suggest that the frequency of IE due to *E. coli* may be higher than previously believed. Because of the high in-hospital mortality and high rates of cardiac surgery in patients with IE due to non-HACEK gram-negative bacilli [1], physicians should pay close attention to the clinical course of patients with *E. coli* IE during hospitalization. In addition, although the *E. coli* detected from the blood culture samples in the present case showed susceptibility to both ABPC and GM, administration of both antibiotics did not prevent the onset of purulent spondylitis. Notably, the purulent spondylitis developed 3 weeks after hospitalization. This may reflect insufficient transferability of antibiotics because it takes 3 to 4 weeks for the formation of new blood vessels in infected bone to accomplish appropriate antibiotic migration [9].

### Patients’ backgrounds

To the best of our knowledge, 32 cases of *E. coli* IE have been reported in the PubMed database in the past 30 years [10–32]. In total, 33 cases (the 32 previously reported cases and the present case) were reviewed in this study (Table 2). These cases included 13 male and 16 female patients; the sex of 4 patients was unknown. The mean age of the patients was 59.6 ± 19.8 years (male, 52.4 ± 21.2 years; female, 64.3 ± 17.2 years). The MV was the most frequently affected structure (15 cases, including 5 cases of prosthetic valve endocarditis) [11, 13, 16, 17, 20, 22, 25, 27, 30, 32], and the aortic valve (AV) was the second most frequently affected structure (11 cases, including 4 cases of prosthetic valve endocarditis) [12, 14–16, 23, 24, 30, 31]. Both the MV and AV were affected in one patient [28]. Non-valvular IE was reported in two patients [26, 29]. The tricuspid or pulmonary valves were also affected; vegetations on the tricuspid valve and pulmonary valve were reported in three and one patient, respectively [1, 18, 19, 21].

**Table 2:** *Escherichia coli* endocarditis cases in the past 30 years

Case No.	First author, year	Age, years	Sex	Comorbidities	Pre-ceding UTI	Complications during hospitalization	Antibiotic therapy	Site of vegetation	Cardiac surgery	Outcome
1	Murray [10] 1988	25	M	None	–	Pulmonary infarction	PC + GM+ MNZ	PV	Resection of anterior PV leaflet	A
2	Watanakunakorn [11] 1992	79	F	DM, HT	–	Hemorrhagic CI, CHF	CFX→ CAZ + GM→CZX	MV-anterior leaflet	–	D (37 days)
3	Raymond [12] 1992	52	F	None	+ *E. coli* positive	Abscess of IVS, CHF	CTRX	AV	–	D (8 days)
4	Oosterbosch [13] 1996	69	F	Multiple myeloma	+ *E. coli* positive	LV aneurysm formation, severe MR, CHF	Temocillin→CXM + AMK	MV-posterior leaflet	–	D (8 days)
5	Morrison [14] 1997	47	M	Multiple tooth decay, chronic alcoholism, bicuspid AV	+*E. coli* positive	Renal infarction	CTRX	AV-RCC	AVR (MP)	A
6	Soma [15] 2005	42	M	AS and AR, urethral stenosis	+*E. coli* positive	Aortic paravalvular abscess formation	TMP-SMX→CTX + GM→ABPC+netilmicin	AV, AV ring abscess	AVR (MP)	A
7	Branger [16] 2005	60	F	Colic polyposis, AF, history of RF, IE, and post-AVR (BP)	–	Aortic regurgitation, CHF	AMPC/CVA + MNZ→AMPC+MNG + GM→CAZ + CPFX	AV (BP)	AVR (MP)	A
8	Branger [16] 2005	75	F	DM, HT, bilateral pyelonephritis, vesicular stenosis	+ *E. coli* positive	Cerebral infarction, mitraltendon rupture, renal failure	CTRX+OFLX	MV	MV annuloplasty	A
9	Branger [16] 2005	76	M	RF, MS→ Post-MVR (MP), colic diverticulosis, kidney cancer	–	Renal failure	AMPC/CVA + CPFX	MV (MP)	–	A
10	Branger [16] 2005	80	M	Ischemic cardiomyopathy, stroke, heart failure	–	Renal failure, *Coxiella burnetii* serology-positive, humeral osteomyelitis	AMPC+GM + DOXY+hydroxychloroquine	AV	–	D (1 month)
11	Branger [16] 2005	66	F	MS→post-MVR (MP)	+*E. coli* positive	Renal failure, MV ring abscess	IPM + GM→CTRX	MV (MP), MV ring abscess	MVR (BP)	A
12	Branger [16] 2005	82	F	DM, thyroid neoplasm	+ *E. coli* positive	Abscess formation, MV ring abscess, perforation, pneumonia	CTRX+OFLX→AMPC+GM	MV, MV ring abscess	MVR (BP)	A
13	Branger [16] 2005	76	F	RF, post-MVR (BP)	+	Renal failure, mitral regurgitation	Unknown	MV (BP)	Unknown	Unknown
14	Kulas [17] 2006	16	F	Puerperal onset after a cesarean section, MR	–	–	AMPC/CVA+ GM + MNZ+ oral CLDM	MV	–	A
15	Foley [18] 2010	31	F	–	–	Tricuspid regurgitation, septic emboli in the lung	MEPM+MNZ→Oral AMPC→IPM + GM	TV anterior leaflet	Resection of anterior TV leaflet	A
16	Tsutsumi [19] 2010	60	M	End-stage alcoholic liver disease, blunt injury of the left arm	–	Osteomyelitis of the left sacroiliac and right sternoclavicular joints, septic emboli in the lung	CTRX→MEPM→CTRX	TV anterior leaflet	–	A
17	Lauridsen [20] 2011	67	M	Acute acalculous cholecystitis	+*E. coli* positive	MR, valve perforation, LV pseudoaneurysm formation, CHF, spondylodiscitis, endophthalmitis	CXM + GM + MNZ→MEPM+GM	MV posterior leaflet	MVR (MP)	A
18	Fordyce [21] 2011	72	M	Post-biopsy of the prostate, sciatica, PAF, AS, MS, MR	+ *E. coli* positive	Transient complete atrioventricular block *ESBL-producing *E. coli* infection	CTRX→ Ertapenem→ IPM + GM	TV septal leaflet	–	A
19	Modi [22] 2011	62	F	MR→Post-MVR (BP), old CI, PC allergy	–	- *ESBL-producing *E. coli* infection	AZT + VCM→IPM	MV (BP)	–	A
20	Senel [23] 2012	60	M	Post-AVR (MP), post-hemorrhoidectomy	Unknown	First-degree atrioventricular block, pseudomembranous enteritis, AV ring abscess	SBT/ABPC→AMPC/CVA→SBT/ABPC+GM	AV (MP), AV ring abscess	AVR (MP)	A
21	Lupse [24] 2012	75	F	Post-cholecystectomy, biliary stenosis and stenting, AR, MR	–	-*ESBL-producing *E. coli* infection	SBT/ABPC→IPM	AV	–	A
22	Rangarajan [25] 2013	54	M	DM, HT, Post-meatotomy and circumcision	+	Severe MR, TIA, CHF, respiratory distress, renal dysfunction	SBT/CPZ + AMK→SBT/CPZ + LVFX	MV anterior leaflet	MVR (MP)	A
23	Gupta [26] 2013	0	M	PDA, VSD, trisomy 18	–	Bilateral hydroureteronephrosis	ABPC+GM→unknown	SVC-RA junction	–	A
24	Spaleniak [27] 2015	76	F	Goodpasture’s syndrome	Unknown	Renal failure *ESBL-producing *E. coli* infection	IPM + CPFX→IPM + AMK	MV anterior leaflet	–	A
25	Chen [28] 2015	46	F	Severe AR, bicuspid AV	+	Severe pulmonary hypertension, CHF	TAZ/PIPC +GM→CAZ + GM	AV and MV anterior leaflet	–	A
26	Tsai [29] 2015	40	M	IgA nephropathy, CRF on HD	Unknown	Necrotizing fasciitis, septic shock, intracranial hemorrhage	CAZ + VCM	LV details unknown	–	D (3 weeks)
27	Loubet [30] 2015	53	Unknown	DM, recent invasive procedure	+	-*ESBL-producing *E. coli* infection	IPM + AMK	AV	–	A
28	Loubet [30] 2015	82	Unknown	Post-AVR (BP), recent invasive procedure	+	Intracardiac abscess	Unknown	AV (BP)	+Details unknown	A
29	Loubet [30] 2015	74	Unknown	Post-MVR (MP)	–	Intracardiac abscess, systemic embolization	CTRX+AMK	MV (MP)	+Details unknown	A
30	Loubet [30] 2015	74	Unknown	DM, post-AVR (MP)	+	Intracardiac abscess	CTX + OFLX	AV (MP)	+Details unknown	D (9 months)
31	Menon [31] 2017	58	M	Post-renal transplant	+ *E. coli* positive	–	CTRX+AMK	AV	–	A
32	Kim [32] 2018	80	F	Thickened MV leaflets, DM, HT, history of pyelonephritis due to *E. coli*	+*E. coli* positive	Multiple cerebral and cerebellar infarctions, emphysematous IE *ESBL-producing *E. coli* infection	MEPM	MV annulus	–	D (39 days)
33	Akuzawa 2018	58	F	Alcohol abuse	–	Purulent spondylitis	SBT/ABPC +GM→MEPM+GM	MV anterior leaflet and chordae tendineae	–	A

Abbreviations: *A* alive, *ABPC* aminobenzylpenicillin, *AMPC* amoxicillin, *AF* atrial fibrillation, *AMK* amikacin, *AS* aortic stenosis, *AR* aortic regurgitation, *AV* aortic valve, *AVR* aortic valve replacement, *AZT* aztreonam, *BP* biological prosthesis, *CAZ* ceftazidime, *CFX* cefoxitin, *CHF* congestive heart failure, *CI* cerebral infarction, *CLDM* clindamycin, *CPFX* ciprofloxacin, *CPZ* cefoperazone, *CRF* chronic renal failure, *CTRX* ceftriaxone, *CTX* cefotaxime, *CVA* clavulanate, *CXM* cefuroxime, *CZX* ceftizoxime, *D* dead, *DM* diabetes mellitus, *DOXY* doxycycline, *ESBL* extended-spectrum β-lactamase, *F* female, *GM* gentamicin, *HD* hemodialysis, *HT* hypertension, *IE* endocarditis, *IVS* interventricular septum, *IPM* imipenem, *LV* left ventricle, *M* male, *MNZ* metronidazole, *MEPM* meropenem, *MP* mechanical prosthesis, *MR* mitral regurgitation, *MS* mitral stenosis, *MV* mitral valve, *MVR* mitral valve replacement, *OFLX* ofloxacin, *PAF* paroxysmal atrial fibrillation, *PC* penicillin, *PDA* patent ductus arteriosus, *PIPC* piperacillin, *PV* pulmonary valve, *RA* right atrium, *RCC* right coronary cusp, *RF* rheumatic fever, *SBT* sulbactam, *SVC* superior vena cava, *TAZ* tazobactam, *TMP-SMX* trimethoprim-sulfamethoxazole, *TV* tricuspid valve, *UTI* urinary tract infection, *VCM* vancomycin, *VSD* ventricular septal defect. *ESBL-producing E. coli infection (highlighted by underlining)

### Background comorbidities of *E. coli* IE

With respect to the comorbidities of *E. coli* IE, seven patients were diagnosed with diabetes mellitus [11, 16, 25, 30, 32], three had a history of malignancy [13, 16], and, notably, three had a history of excessive alcohol consumption [14, 19]. Moreover, three patients had renal disease [27, 29, 31]. Two patients required renal replacement therapy [27, 29], including one patient taking prednisolone and cyclophosphamide [27], and one patient had undergone renal transplantation [31]. Conditions such as diabetes, hemodialysis, and malignancy may reflect an underlying immunocompromised state. A past study showed an increasing incidence of non-HACEK gram-negative endocarditis in patients with cirrhosis [33]; interestingly, however, our review included three patients with a history of excessive alcohol consumption with or without cirrhosis. Chronic alcohol consumption causes disintegrity of the gut mucosa, which may lead to an increased risk of transmural migration of *E. coli* into the circulation and non-oral gastrointestinal tract. Commonly, the urinary tract is presumed to be a source of pathogen acquisition in patients with non-HACEK gram-negative endocarditis [33, 34]. In addition to this, excessive alcohol consumption may be an independent risk factor for *E. coli* IE. Patients undergoing renal replacement therapy also have a high incidence of endocarditis [35]; therefore, it should be noted that these comorbidities (diabetes, malignancy, excessive alcohol consumption, and hemodialysis) may be risk factors for *E. coli* IE. Preceding urinary tract infection was reported in 52% (17/33) of the patients [12–15, 20, 21, 25, 28, 30–32]. Additionally, 36% (12/33) of the patients were positive for *E. coli* in urine culture samples [12–15, 20, 21, 25, 31, 32].

### Prosthetic valve endocarditis and native valve endocarditis

Endocarditis due to non-HACEK gram-negative bacteria was considered a disease of intravenous drug users in the past, but the reported frequency of this type of endocarditis in intravenous drug users is no more than 4% of all affected patients [33]. Our review included no intravenous drug users. Instead, *E. coli* IE is more common in patients with prosthetic valves; 33% (5/15) of the affected MVs and 36% (4/11) of the affected AVs were prosthetic valves (Table 3). This incidence of *E. coli* IE associated with prosthetic valves is slightly higher than the previously reported incidence (7–25%) [36]. A recent analysis of the International Collaboration on Endocarditis–Prospective Cohort Study (ICE-PCS) registry showed a rising incidence of non-HACEK gram-negative endocarditis in patients with implanted endovascular devices, including prosthetic valves, permanent pacemakers, and implantable cardioverter-defibrillators [33], and this may be applicable to patients with *E. coli* IE. However, 67% (22/33) of reviewed patients developed native valve *E. coli* IE [10–21, 24, 25, 27, 28, 30–32]. Among these 22 patients, degenerative valvular diseases were reported in 7 patients, accounting for no more than 32% (7/22) of cases of native valve *E. coli* IE. Conversely, 68% (15/22) of patients with native valve *E. coli* IE had no history of degenerative valvular disease. This contrasts with IE caused by common pathogens including streptococci, staphylococci, and enterococci because about 70% of such patients have degenerative valvular disease [37]. The reason why normal valves are more frequently affected in *E. coli* IE is unclear. In the present review, 52% (17/33) of patients had a urinary tract infection prior to or at the time of diagnosis of *E. coli* IE [12–16, 20, 21, 25, 28, 30–32]. Notably, 12 of these 17 patients were positive for *E. coli* in both the urine culture and blood culture, and 13 of the 17 patients had native valve IE [12–16, 20, 21, 25]. This suggests that virulence factors of extraintestinal pathogenic *E. coli* existing in the urinary tract may be strongly associated with the onset of *E. coli* IE. Notably, recent studies have indicated that extraintestinal pathogenic *E. coli* strains have a high prevalence of phylogenetic type B2, which has a variety of virulence factors that may enable the pathogens to invade cardiac endothelia [38, 39]. Moreover, the ICE-PCS registry reported that it takes ≥1 month from onset to clinical diagnosis in 90% of patients with non-HACEK gram-negative endocarditis [33]. This suggests that a prolonged duration of insufficient treatment resulting from a poor understanding of *E. coli* IE may increase the opportunity for *E. coli* to invade the endothelia of normal valves. Importantly, the initial foci of *E. coli* infection were unclear in 48% (16/33) of the reviewed cases, including our case. Therefore, a list of differential diagnoses for *E. coli* IE should be properly compiled when examining patients with *E. coli* bacteremia.

**Table 3:** Characteristics of prosthetic valve and native valve endocarditis due to *E. coli*

	Prosthetic valve endocarditis (*n* = 9)	Native valve endocarditis (*n* = 22)
*Patients’ characteristics*		
Age, years	70.0 ± 7.65	59.7 ± 18.3
Sex		
Male	2	13
Female	4	8
Unknown	3	1
*Infected valves*		
Aortic valve	4	7
Mitral valve	5	10
Aortic + mitral valves	0	1
Tricuspid valve	0	3
Pulmonary valve	0	1
*Types of prosthetic valves*		
Aortic valve		
Biological	2	–
Mechanical	2	–
Mitral valve		
Biological	2	–
Mechanical	3	–
*Preexisting valvulopathy in native valves*		
Aortic valve	–	3
Mitral valve	–	2
Aortic + mitral valves	–	2
Tricuspid valve	–	0
Pulmonary valve	–	0

Two patients with nonvalvular endocarditis were excluded

### Complications during hospitalization

Peripheral embolization, congestive heart failure, and valve-ring abscesses have been reported as complications of non-HACEK gram-negative endocarditis [33]. Among all reviewed patients in the present report, eight (24%) had peripheral embolization [10, 11, 14, 16, 18, 19, 30, 32], and six (22%) had congestive heart failure [11–13, 16, 20, 25]. Intracardiac or valve-ring abscesses were reported in eight (18%) patients [12, 15, 16, 23, 30]. Infective myocarditis leading to left ventricular aneurysm formation was reported in two (6%) patients [13, 20]. Interestingly, newly observed atrioventricular block was reported in two (6%) patients: one with tricuspid valve endocarditis and the other with AV endocarditis and a ring abscess [21, 23]. These findings may suggest that potent invasive myocardial infection by *E. coli* leading to destruction of the myocardium can occur before detection of echocardiographic abnormalities. Renal failure was also reported in six (18%) patients [16, 25, 27]. Moreover, suppurative osteomyelitis or spondylodiscitis was observed in four (12%) patients [16, 19, 20]. Physicians should know the necessity of evaluating patients for orthopedic complications when treating them for *E. coli* IE.

### Treatment and outcome

The American Heart Association recommends combination antibiotic therapy with β-lactams (penicillins, cephalosporins, or carbapenems) and either an aminoglycoside or fluoroquinolone for 6 weeks in patients with non-HACEK gram-negative endocarditis [5]. Among 31 patients in whom the antibiotics used could be identified, 6 (20%) were treated with penicillins [10, 16, 17, 23, 26], 14 (45%) were treated with cephems and/or penicillins [11–16, 25, 28–31], and 11 (35%) were treated with carbapenems and/or other β-lactams [16–22, 24, 27, 30–32]. Although only six patients were positive for extended-spectrum β-lactamase (ESBL)-producing *E.coli* [21, 22, 24, 27, 30, 32], it should be noted that one-third of the reviewed patients were treated with carbapenems. Notably, five of the six ESBL-positive patients were successfully treated through the administration of carbapenems without surgical interventions. This may suggest the importance of prompt administration of carbapenems when needed because *E. coli* may potentially have resistance against β-lactams such as inducible β-lactamases, which can be overlooked in routine in vitro laboratory screening for antibiotic susceptibility [5]. Therefore, antibiotic stability against β-lactamases should be considered when choosing β-lactams for therapy of *E. coli* IE. Aminoglycosides and fluoroquinolones were administered with β-lactams or carbapenems in 21 and 7 patients, respectively [10, 11, 13, 15–18, 20, 21, 23, 25–28, 30, 31]. Four patients were treated with both aminoglycosides and fluoroquinolones besides β-lactams or carbapenems, and three of these four patients required surgical treatment despite ESBL-negative *E. coli* infection [16, 25, 27]. This suggests the importance of a thorough evaluation of patients with *E. coli* IE to assess the necessity of surgical intervention.

Among patients with non-HACEK gram-negative endocarditis, the mortality rate did not significantly differ between patients receiving medical therapy alone and those undergoing surgical treatment, and the in-hospital mortality and cardiac surgery rates were reportedly 24% and 51%, respectively [33]. Among these reviewed patients with *E. coli* IE, the mortality and surgical intervention rates were 21% (7/33) and 42% (14/33), respectively [10–16, 18, 20, 23, 25, 29, 30, 32]. Almost half of the patients (4/7) with fatal *E. coli* IE died within 1 month of hospitalization [11–13, 16, 29]. Meanwhile, the 14 patients who underwent surgical intervention included six (43%) patients with prosthetic valve endocarditis and eight (57%) patients with native valve endocarditis. Prosthetic valve replacement was performed in eight patients (four AV and four MV replacements) [14–16, 20, 23, 25], and MV annuloplasty was performed in one patient [16]. Resection of the infected valve leaflets without valve replacement was performed in two patients and was mainly done only in patients with tricuspid or pulmonary valve endocarditis [10, 18]. In three patients, details of the surgical procedure were unclear [30]. One patient died 9 months later [30], but in-hospital postoperative death was not reported among the reviewed patients, which may suggest the importance of the timing of the decision regarding surgical intervention. Limitations of our report include the lack of detailed observation of the patients’ MV on transesophageal echocardiography because of the patients’ refusal and the inability to statistically investigate the data of reviewed patients with *E. coli* IE because this report was based on a limited number of case reports. However, our findings suggest important characteristics of *E. coli* IE.

## Conclusion

Our review shows that native valves without degenerative valvulopathy can be more susceptible to *E. coli* IE than prosthetic valves in patients with risk factors such as diabetes mellitus, malignancy, excessive alcohol consumption, and renal replacement therapy. Peripheral embolization, congestive heart failure, and valve-ring abscesses are major complications of *E. coli* IE; however, ventricular aneurysm and atrioventricular block resulting from infective myocarditis, which are less frequent complications, should also be noted. Cephems and carbapenems are frequently used for treatment, and the mortality and surgical intervention rates associated with this therapy are 21% and 42%, respectively. Physicians should be cognizant of the timing of surgical interventions when *E. coli* IE is resistant to antibiotic therapy.

## Abbreviations

AV: Aortic valve
CRP: C-reactive protein
*E. coli*: *Escherichia coli*
ESBL: Extended-spectrum β-lactamase
GM: Gentamycin
HACEK: *Haemophilus* spp., *Aggregatibacter* spp., *Cardiobacterium hominis*, *Eikenella corrodens*, and *Kingela* spp.
ICE-PCS: International Collaboration on Endocarditis–Prospective Cohort Study
IE: Infective endocarditis
MRI: Magnetic resonance imaging
MV: Mitral valve
SBT/ABPC: Sulbactam/ampicillin
